# Macrophage-Colony Stimulating Factor Derived from Injured Primary Afferent Induces Proliferation of Spinal Microglia and Neuropathic Pain in Rats

**DOI:** 10.1371/journal.pone.0153375

**Published:** 2016-04-12

**Authors:** Masamichi Okubo, Hiroki Yamanaka, Kimiko Kobayashi, Yi Dai, Hirosato Kanda, Hideshi Yagi, Koichi Noguchi

**Affiliations:** 1 Department of Anatomy and Neuroscience, Hyogo College of Medicine, Nishinomiya, Hyogo 663–8501 Japan; 2 Department of Pharmacy, School of Pharmacy, Hyogo University of Health Sciences. Kobe, Hyogo 650–8530, Japan; Toronto University, CANADA

## Abstract

Peripheral nerve injury induces proliferation of microglia in the spinal cord, which can contribute to neuropathic pain conditions. However, candidate molecules for proliferation of spinal microglia after injury in rats remain unclear. We focused on the colony-stimulating factors (CSFs) and interleukin-34 (IL-34) that are involved in the proliferation of the mononuclear phagocyte lineage. We examined the expression of mRNAs for macrophage-CSF (M-CSF), granulocyte macrophage-CSF (GM-CSF), granulocyte-CSF (G-CSF) and IL-34 in the dorsal root ganglion (DRG) and spinal cord after spared nerve injury (SNI) in rats. RT-PCR and *in situ* hybridization revealed that M-CSF and IL-34, but not GM- or G-CSF, mRNAs were constitutively expressed in the DRG, and M-CSF robustly increased in injured-DRG neurons. M-CSF receptor mRNA was expressed in naive rats and increased in spinal microglia following SNI. Intrathecal injection of M-CSF receptor inhibitor partially but significantly reversed the proliferation of spinal microglia and in early phase of neuropathic pain induced by SNI. Furthermore, intrathecal injection of recombinant M-CSF induced microglial proliferation and mechanical allodynia. Here, we demonstrate that M-CSF is a candidate molecule derived from primary afferents that induces proliferation of microglia in the spinal cord and leads to induction of neuropathic pain after peripheral nerve injury in rats.

## Introduction

Microglia colonizes the central nervous system during development, originating from hematopoietic stem cells found in yolk sac [1]and accounts for 5–20% of the glial cell population in the adult brain [2]. Microglia play a role in both physiological and pathological conditions. For example, Paolice et al. demonstrated that microglia are involved in synaptic pruning of excessive synapses in the developing brain [3]. Moreover, increasing evidence suggests that microglia are able to survey the microenvironments of the adult brain to check whether there is an emergency event. Once pathological conditions are detected, microglia accumulates, activates and proliferates within the affected lesion immediately, and starts to clear the debris and to repair the tissues. In terms of microglial accumulation and activation in pathological conditions, many studies suggested that purinergic signaling is a key inducer of these phenotypes. *In vitro*, microglia showed chemotaxis against ATP, and migrated towards sites of higher concentrations of ATP [4]. Phosphorylation of p38 mitogen activated protein kinase (p-p38 MAPK), one of the indicators for activated-state, occurred in microglia following application of ATP *in vitro* [5]. Moreover, we demonstrated that peripheral nerve injury increases p-p38 in activated microglia *via* the P2Y12 receptor, an ATP receptor, leading to neuropathic pain [6]. Previous reports have strongly suggested that accumulation and activation of microglia in an injured segment of the spinal cord are involved in neuropathic pain after peripheral nerve injury. In contrast, the precise mechanisms of microglial proliferation are largely unknown. Furthermore, it remains unclear what molecules derived from injured-primary afferents work for the proliferation of microglia in the spinal cord and neuropathic pain following peripheral nerve injury.

Colony stimulating factors (CSFs) are cytokines that are involved in the proliferation and differentiation from hematopoietic stem cells to a specific kind of white blood cells, such as macrophages and granulocytes [7]. Three types of CSFs, macrophage-colony stimulating factor (M-CSF), granulocyte macrophage-colony stimulating factor (GM-CSF) and granulocyte-colony stimulating factor (G-CSF) have been shown to exist [8–10], and these CSFs each have specific receptors; M-CSF receptor (M-CSFr), GM-CSF receptor alpha (GM-CSFr alpha) and G-CSF receptor (G-CSFr) [11–13]. Recently, IL-34 was identified a new ligand for M-CSFr and has similar roles with M-CSF [14, 15].

Elmore et al. demonstrated that continuous administration of a M-CSFr inhibitor depleted microglia in the brain, and then repopulation of microglia occurred after discontinuation of the administration of the M-CSFr inhibitor [16]. Facial nerve injury increased M-CSF expression in microglia around the facial nucleus and M-CSF activated the cycling dependent kinase family prior to the proliferation of microglia *in vitro* [2, 17]. These reports suggested that M-CSF has a pivotal role in survival, differentiation and proliferation of microglia *in vitro* [18].

In the very recent past, Guan et al. demonstrated that M-CSF produced by injured primary afferent is involved in microglial proliferation in the spinal cord and in neuropathic pain after peripheral nerve injury in mice [19]. Herein, we examined whether induction of M-CSF in injured-primary afferent neurons contributes to the proliferation and activation of spinal microglia *in vivo* and that M-CSF/M-CSFr signaling leads to generation of neuropathic pain after peripheral nerve injury using different methods and species that were used by Guan et al [19].

## Materials and Methods

### Animal procedures

Male Sprague Dawley rats (200–250 g) were anesthetized with sodium pentobarbital (50 mg/kg, i.p.) and the tibial and common peroneal nerves were transected, while the sural nerve was left intact (spared nerve injury; SNI model) [20]. The wounds were closed, and the rats were allowed time to recover in the cage. At several time points (0, 0.5, 1, 2, 3, 7 and 14 d) after the SNI, groups of rats were processed for histological analysis (n = 4–5 at each time point). Peripheral tissue inflammation was introduced by an intraplantar injection of 100 μl of complete Freund’s adjuvant (CFA, Sigma-Aldrich, Saint Louis, MO) suspended in an oil/saline (1: 1) emulsion. Every effort and care was made to minimize animal suffering during surgery and post-surgical periods including maintenance of body temperature, and reduce the number of animals used. The animals were housed in polycarbonate cages (W43 x H 28 x D 21 cm) with a deep layer of saw dust, 3 animal in each cage, in a thermo-statically controlled room at 25.0 ± 1.0°C. The room was artificially illuminated from 8:00 a.m. to 8:00 p.m. The animals received commercial pelleted rat feed (CE-2, CLEA JAPAN INC.) and water *ad libitum*.

All animal experimental procedures were approved by the Hyogo College of Medicine Committee on Animal Research (Permit Number: 13–048) and were performed in accordance with the National Institutes of Health guidelines on animal care.

### Reverse transcription-polymerase chain reaction (RT-PCR) and in situ hybridization histochemistry (ISHH)

The rats were killed by decapitation under deep ether anesthesia. The ipsilateral side of spinal cords (L4-L5) and DRGs (L4-5) were removed and rapidly frozen with powdered dry ice and stored at -80°C until use. Extraction of total RNA was done using a single step extraction method with ISOGEN (Nippon Gene, Tokyo, Japan) as described in a previous paper [21]. PCR primers for CSFs, IL-34 and M-CSFr and glyceraldehyde 3-phosphate dehydrogenase (GAPDH) cDNA were designed as shown in Table 1. PCR products were used to generate the cRNA probes for ISHH. The L4-L5 spinal cord and ipsilateral DRGs were cut on a cryostat at a 12 and 5 μm thickness, respectively. The protocol for ISHH was described in detail previously [22]. Briefly, a clone (pCRII-TOPO, Invitrogen, Carlsbad, CA) containing a partial sequence corresponding to the coding regions of CSFs, IL-34 and their receptor were prepared. Using the enzyme-digested clones, ^35^S UTP-labeled antisense and sense cRNA probes were synthesized. The ^35^S-labeled probes in hybridization buffer were placed on the section, and then incubated at 55°C overnight (>18 hours). Sections were then washed and treated with 1 μg/ml RNase A. Subsequently, sections were dehydrated and air-dried. After the hybridization reaction, the slides were coated with NTB emulsion (Kodak, Rochester, NY, USA) and exposed for 4–8 weeks. Once developed in D-19 (Kodak), the sections were stained with hematoxylin-eosin and coverslipped.

**Table 1:** Sequence location of primers.

Gene	Acc. No.	Forward	Reverse	Length (bp)
M-CSF	NM_023981	1731–1750	2205–2186	474
GM-CSF	NM_053852	42–61	426–407	384
G-CSF	NM_017104	628–647	1159–1140	531
IL-34	NM_001025766	180–199	704–85	524
M-CSFr	NM_001029901	2571–2590	3075–3056	504
GAPDH	M17701	80–99	350–331	270

### Immunohistochemistry (IHC)

Rats were deeply anesthetized with sodium pentobarbital and perfused transcardially with 250 ml of 1% formaldehyde (FA) in 0.1 M phosphate buffer (PB), pH 7.4, followed by 500 ml of 4% FA in 0.1 M PB. The spinal cords and DRGs were dissected out and postfixed in the same fixative at 4°C overnight, followed by immersion in 20% sucrose in 0.1 M PB at 4°C for 2 days. The tissue was frozen in powdered dry ice and cut on a cryostat at a 16 μm thickness for the spinal cord. The sections were processed for IHC using the ABC method [23]. The following antibodies were used: rabbit anti-activating transcription factor 3 (ATF3) polyclonal antiserum (1: 500; Santa Cruz, University of California, San Francisco, CA), rabbit anti-ki67 polyclonal antiserum, (1: 2500; Abcam, Cambridge, MA), rabbit anti-ionized calcium-binding adapter molecule 1 (Iba1) polyclonal antiserum (1:200; Wako Chemicals, Tokyo, Japan), goat anti-Iba1 polyclonal antiserum (1:1000; Abcam, Cambridge, MA), rabbit anti-phospho-p38 MAPK (p-p38) polyclonal antiserum (1: 500; Cell Signaling Technology, Beverly, MA), mouse anti-neuronal specific nuclear protein (NeuN) monoclonal antiserum (1:1000; Chemicon, Temecula, CA), and rabbit anti-glial fibrillary acidic protein (GFAP) polyclonal antiserum (1:1000; Dako Cytomation, Glostrup, Denmark). In brief, spinal cord sections were incubated with a primary antibody overnight at 4°C, followed by biotinylated secondary antibodies (1: 500; Vector Laboratories, Vector Laboratories, CA) overnight at 4°C. Antibodies were visualized by 0.05% 3,3-diaminobenzidine tetrahydrochloride (Wako Chemicals). Double-immunofluorescent staining was performed with anti-rabbit Alexa Fluor 594 IgG (1:1000; Invitrogen, San Diego, CA), anti-goat Alexa Fluor 488 IgG (1:1000), and anti-mouse Alexa Fluor 488 IgG (1:1000) after incubation with respective primary antibodies. The detail treatment of sections and methods of double labeling with IHC and ISHH were described previously [24]. For quantification, a box measuring 1.35 x 10^5^ μm^2^ was placed onto areas of the medial 2/3 of the dorsal horn (lamina II-III) under x20 magnification using a microscope, and the number of profiles that were positive for each maker was counted in this area. These measurement protocols were performed on 8–10 spinal sections from each animal.

### Photomicrographs

All DAB stained and emulsion coated slides were digitized with a Nikon Eclipse 80i microscope connected to a Nikon DXM-1200F digital camera (Nikon Corporation, Tokyo, Japan). Immunofluorescence images were taken by the Olympus FLUOVIEW FV1200 confocal microscope (Olympus Corporation, Tokyo, Japan). A maximum of 21 images of confocal z stacks were sectioned at 8 μm. Z projection of stacks series was averaged using ImageJ (1.46r). Adobe Photoshop CS4 (Adobe Systems, Mountain View, CA) was used to optimize the images and compose all figures.

### Drug treatments

At the same time as SNI surgery, the L5 vertebra was laminectomized under adequate anesthesia with sodium pentobarbital, and a 7 cm soft tube (SILASTIC laboratory tubing, Dow Corning Corporation, MI; outer diameter, 0.64 mm) filled with saline was inserted into the subarachnoid space for an ~0.5 cm length. After the incision was closed, the tube was fixed, and the cut end was ligated. Then, the tube was laid under the skin and the incision was closed. M-CSF receptor inhibitor GW2580 (5-(3-Methoxy-4-((4-methoxybenzyl)oxy)benzyl)-pyrimidine-2,4-diamine)(Calbiochem, San Diego, CA) and rat recombinant M-CSF (Sigma-Aldrich, Saint Louis, MO) were carefully injected in a volume of 10 μL followed by 5 μL saline. The concentrations of GW2580 was 10 nmol diluted in dimethyl sulfoxide (DMSO), M-CSF was 10 μg diluted in saline. An adequate concentration of DMSO and BSA were used as a vehicle control. The M-CSF inhibitor was given 3 times at the same time as SNI surgery, 25 and 40 hours after SNI. Rat recombinant M-CSF was given twice at a 24 h interval. Rats were anesthetized and drugs were injected using a Hamilton syringe.

### Behavioral testing

All rats were tested for mechanical allodynia on the plantar surface of the hindpaw in pretreatment (0), 1 and 2 days in each experiment. Mechanical allodynia was assessed with a dynamic plantar anesthesiometer (Ugo Basile, Comerio, Italy) [25]. Detailed measurement of mechanical threshold in the rat hindpaw was described in our previous report [6].

### Image analysis

Measurements of the density of silver grains over randomly selected tissue profiles were performed using a computerized image analysis system (NIH Image, version 1.61), where only neuronal profiles that contained nuclei were used for quantification. At a magnification of 200× and with bright-field illumination, upper and lower thresholds of gray level density were set such that only silver grains were accurately discriminated from the background in the outlined cell or tissue profile and read by the computer pixel-by-pixel. Subsequently, the area of discriminated pixels was measured and divided by the area of the outlined profile, giving a grain density for each cell or tissue profile. To reduce the risk of biased sampling of the data because of varying emulsion thickness, we used a signal-to-noise (S/N) ratio for each cell in each tissue. The S/N ratio of an individual neuron and its cross-sectioned area, which was computed from the outlined profile, was plotted. Based on this scatter gram, neurons with a grain density of five-fold the background level or higher (5 < S/N ratio) were considered positively labeled for M-CSF mRNA. Because a stereological approach was not used in this study, quantification of the data may represent a biased estimate of the actual numbers of neurons. At least 500 neurons from the L4/5 DRG of each rat were measured.

### Statistics

Data are expressed as mean ± SEM. Differences in changes of values over time of each group were tested using one-way ANOVA, followed by individual post hoc comparisons (Fisher's exact test) or pairwise comparisons (t test) to assess differences of values between naive versus each time point of the SNI groups. A difference was accepted as significant if *p* < 0.05.

## Results

### Peripheral nerve injury drastically increased the expression of M-CSF in injured DRG neurons

**Fig 1:**
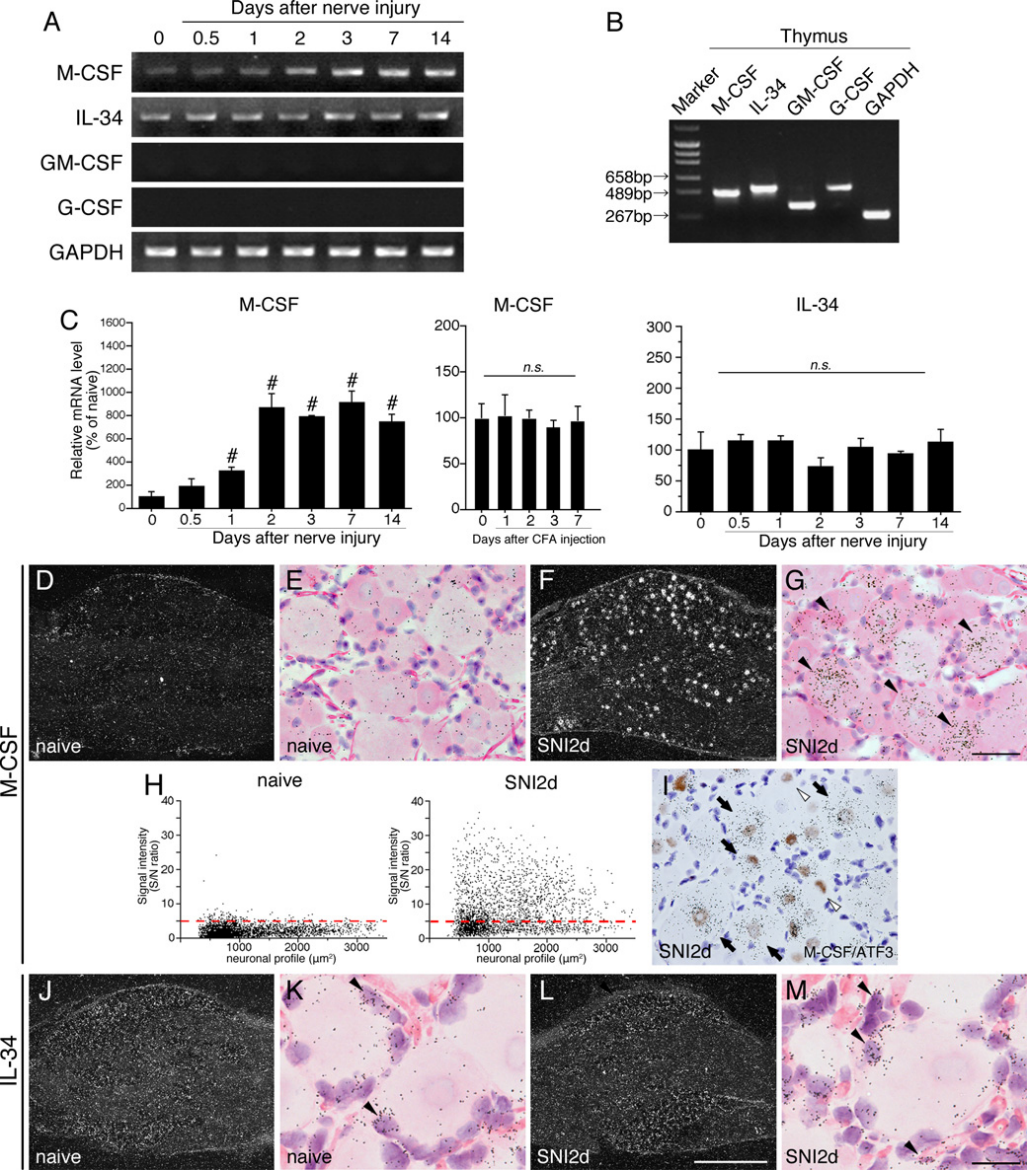
Expression of mRNA for a series of CSFs and IL-34 in DRG after peripheral nerve injury. PCR products from the L4-5 DRG taken from 0 (naive), 0.5, 1, 2, 3, 7, and 14 days after SNI. Representative gel panels are shown in A. (B) Electrophoresis image shows positive control bands for each primer set in thymus. Use of each gene primers result in PCR products of 474 bp (M-CSF), 524 bp (IL-34), 384 bp (GM-CSF), 531bp (G-CSF), and 270 bp (GAPDH), respectively. (C) Graphs show Quantification of the relative mRNA levels of M-CSF (left), IL-34 (right) in DRG after SNI and M-CSF (middle) in DRG after intraplantar injection of CFA. M-CSF and IL-34 mRNA levels were normalized against GAPDH (*n* = 4; mean ± SEM; #, *p* < 0.05 compared with naive). (D-M) Expression pattern of M-CSF and IL-34 mRNAs in DRG after peripheral nerve injury. Darkfield ISHH-images show the expression of M-CSF (D, F) and IL-34 (J, L) mRNAs in DRG of naive (D, J) and 2 days after nerve injury (F, L), respectively. (E, G, K, M) Higher magnification brightfield images of the left-hand photographs. The solid arrowheads indicate samples of the positively labeled cells. Sections were counterstained by H-E. (H) Scatterplot diagrams showing the distribution of M-CSF mRNA in DRGs of naive (left) and 2 days after nerve injury (right). Individual cell profiles are plotted according to the cross-sectional area (um^2^; along the x-axis) and signal intensity (S/N ratio, along the y-axis). The red dashed lines indicate the borderline between the negatively and positively labeled cells (S/N ratio = 5). (I) Photograph shows the double labeling study of ISHH for M-CSF mRNA with immunostaining of ATF3 in DRG at 2 days after nerve injury. Sections were counterstained by hematoxylin. Open arrowheads indicate single immunostained cells (brown staining). Arrows indicate double-labeled cells. Scale bar = 500 μm in D, F, J, L; 25 μm in E, G, I; 12.5 μm in K, M.

We first examined the gene expression levels of CSF receptor ligands, M-CSF, IL-34, GM-CSF and G-CSF in the rat dorsal root ganglion (DRG) after peripheral nerve injury using a conventional RT-PCR method (Fig 1A–1C). We used the L4-5 DRGs which were taken at 0 (naive), 0.5, 1, 2, 3, 7 and 14 days after SNI surgery. M-CSF and IL-34 mRNAs were detected in naive DRGs (Fig 1A). The expression level of M-CSF mRNA dramatically increased in the DRG from 1 to at least 14 days after SNI (Fig 1A, left in C). We next tested the M-CSF mRNA expression level in DRG of inflammatory pain model, complete Freund’s adjuvant (CFA) model, in which only limited activation of spinal microglia [26]. Importantly, M-CSF mRNA had no change in DRG of CFA model rats (middle in Fig 1C). IL-34 mRNA did not change in the DRG after SNI (Fig 1A, right in C). GM- and G-CSF mRNAs could not be detected in the DRG under both the naive and SNI model conditions (Fig 1A). These results led us to examine whether the primers for GM- and G-CSF were available. We confirmed that all primers could generate the precise size of PCR products in rat thymus as positive controls (Fig 1B) and that these PCR fragments were correct by DNA sequencing (data not shown). Information of each primer is shown in Table 1.

To clarify the expression pattern of M-CSF and IL-34 mRNAs in the DRG, we performed *in situ* hybridization histochemistry (ISHH) using radioisotope (RI)-labeled cRNA probes (Fig 1D–1M). As shown in the photographs of ISHH for M-CSF mRNA, very few aggregated silver grains were detected in naive DRG neurons (Fig 1D and 1E) indicating that M-CSF mRNA was faintly expressed in naive DRG neurons. Two days after nerve injury, M-CSF mRNA dramatically increased in DRG neurons (Fig 1F and 1G). Brightfield photomicrographs confirmed the presence of these silver grains over the neuronal profiles whose nuclei were lightly stained with hematoxylin (Fig 1G). To investigate the distribution pattern of M-CSF mRNA, we measured, calculated, and plotted the S/N ratio and cross-sectional area of each neuron (Fig 1H). Neuronal profiles with a grain density of 5-fold the background level or higher (S/N ratio ≧ 5) were considered positively labeled for mRNA in accordance with a previous study [27]. With this criterion, the M-CSF mRNA was positive in 3.9 ± 0.7% of the total neurons in the naive DRG and was positive in 50.3 ± 2.5% of the total neurons of the DRG (day 2). The scatterdiagram revealed that M-CSF mRNA in the naive DRG was faintly expressed in each size neurons (left in Fig 1H). M-CSF mRNA was robustly upregulated and widely distributed through the small neurons to the largest ones after peripheral nerve injury (right in Fig 1H). M-CSF mRNA in the naive DRGs was expressed in 4.5 ± 1.9% in small (<600 μm^2^), 4.4 ± 0.8% in medium-size (600–1200 μm^2^) and 2.4 ± 0.7% in large-sized (>1200 μm^2^) neurons. In SNI model (2 days), M-CSF mRNA was expressed in 40.2 ± 3.5% in small-, 43.0 ± 2.2% in medium- and 60.5 ± 4.1% in large-sized neurons of DRGs, respectively. The definition of neuronal size was described previously [27, 28].

To clarify upregulation of M-CSF mRNA in the DRG was injury-dependent, we combined ISHH for M-CSF mRNA with immunohistochemistry (IHC) with a widely used antibody, activating transcription factor 3 (ATF3), an injured-neuronal maker [29]. Two days after nerve injury, we found ATF3 positive neurons in 58.4 ± 3.2% of the total neurons of the DRG. No specific immunostaining of the antibody was observed in the absence of primary antibodies (data not shown). The coexpression study showed that 96.2 ± 0.4% of M-CSF positive cells were colocalized with ATF3, and conversely, 63.1 ± 1.3% of ATF3 positive cells were colocalized with M-CSF mRNA. M-CSF mRNA was heavily colocalized with ATF3 positive cells after nerve injury (Fig 1I) indicating that induction of M-CSF mRNA was dependent on nerve injury.

The low-magnification darkfield images of ISHH revealed that IL-34 mRNA seemed to be expressed in non-neuronal cells (Fig 1J and 1L). The high-magnification brightfield images of ISHH confirmed that they were exclusively expressed in non-neuronal cells around DRG neurons (arrowheads in Fig 1K and 1M). IL-34 mRNA did not change in DRGs between naive and 2 days after SNI which is consistent with the results of RT-PCR (Fig 1A and 1C). These results indicate that peripheral nerve injury, but not inflammation, predominantly induces M-CSF mRNA expression in injured-DRG neurons and that IL-34 mRNA is constitutively expressed in non-neuronal cells and is not affected by peripheral nerve injury.

### Expression of CSFs and IL-34 in the spinal cord after peripheral nerve injury

**Fig 2:**
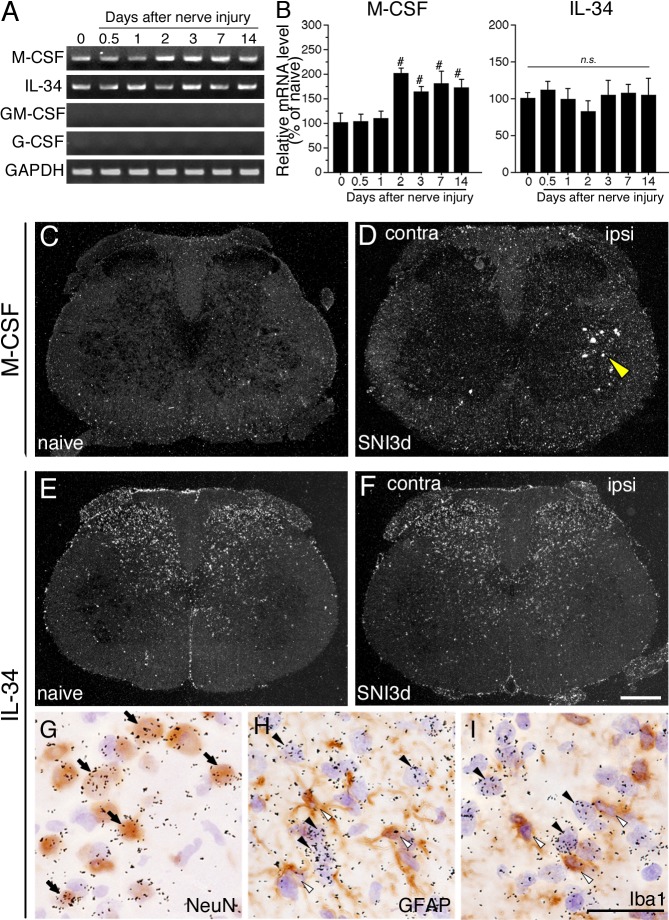
Expression of M-CSF and IL-34 in the spinal cord after peripheral nerve injury. PCR products from the spinal cord (L4-5) taken from 0 (naive), 0.5, 1, 2, 3, 7, and 14 days after surgery. Representative gel panels are shown in A. (B) Graphs show quantification of the relative mRNA levels of M-CSF (left) and IL-34 (right) in spinal cord after SNI. M-CSF and IL-34 mRNA levels were normalized against GAPDH (*n* = 4; mean ± SEM; #, *p* < 0.05 compared with naive). (C-F) Lower power darkfield ISHH-images show the expression of M-CSF (C, D) and IL-34 (E, F) mRNAs in spinal cord (L4-5) of naive (C, E) and 3 days after nerve injury (D, F). Open arrowhead in D indicates the signals of M-CSF mRNA in motoneurons after SNI. (G-I) Double labeling study of ISHH for IL-34 mRNA with immunohistochemistry of NeuN (G), GFAP (H) and Iba1 (I) in the dorsal horn of the spinal cord at 3 days after nerve injury. Arrowheads indicate single-labeled cells by ISHH (aggregation of grains), and open arrowheads indicate single immunostained cells (brown staining). Sections were counterstained by hematoxylin. Arrows indicate double-labeled cells. Scale bar = 500 μm in C-F; 25 μm in G-I. contra, contralateral; ipsi, ipsilateral.

We next asked whether expression of mRNAs for M-CSF, IL-34, GM-, and G-CSF in the spinal cord were affected by peripheral nerve injury using same techniques (Fig 2). RT-PCR revealed that M-CSF mRNA was expressed in the spinal cord of naive rats and increased from 2 to 14 days after nerve injury (Fig 2A and 2B). IL-34 mRNA was constitutively expressed in the spinal cord of naive rats (Fig 2A and 2B). Expression levels of IL-34 mRNA were not affected by nerve injury as shown in Fig 2A and 2B. GM- and G-CSF mRNAs could not be detected in the spinal cord of both naive and SNI model rats (Fig 2A) as well as in their DRGs. We next performed ISHH to investigate the expression patterns of M-CSF and IL-34 mRNAs in the spinal cord following peripheral nerve injury (Fig 2C–2F). Darkfield imaging of ISHH revealed that M-CSF mRNA was slightly expressed throughout the naive spinal cord as shown in Fig 2C, and induced in the ventral horn indicating that M-CSF mRNA was apparently induced in motoneurons on the ipsilateral side (yellow arrowhead in Fig 2D). In contrast to M-CSF mRNA expression, darkfield images of ISHH revealed that IL-34 mRNA was constitutively and widely expressed in the spinal dorsal horn and had no change after nerve injury (Fig 2E and 2F). These results were consistent with RT-PCR (Fig 2A and 2B). We next conducted double labeling analysis with ISHH and IHC in the spinal dorsal horn (Fig 2G–2I). We used several cellular markers for IHC: NeuN, GFAP and Iba1, markers of neuronal nuclei, astrocytes and microglia, respectively. The double labeling study revealed that IL-34 mRNA in the spinal dorsal horn at 3 days after nerve injury was colocalized with NeuN (arrows in Fig 2G), but not GFAP and Iba1-immunoreacitve (ir) cells (Fig 2H and 2I). These results indicated that M-CSF mRNA increases in injured motoneurons and IL-34 mRNA is constitutively expressed in a subset of spinal neurons.

### M-CSFr mRNA was preferentially induced in spinal microglia after peripheral nerve injury

**Fig 3:**
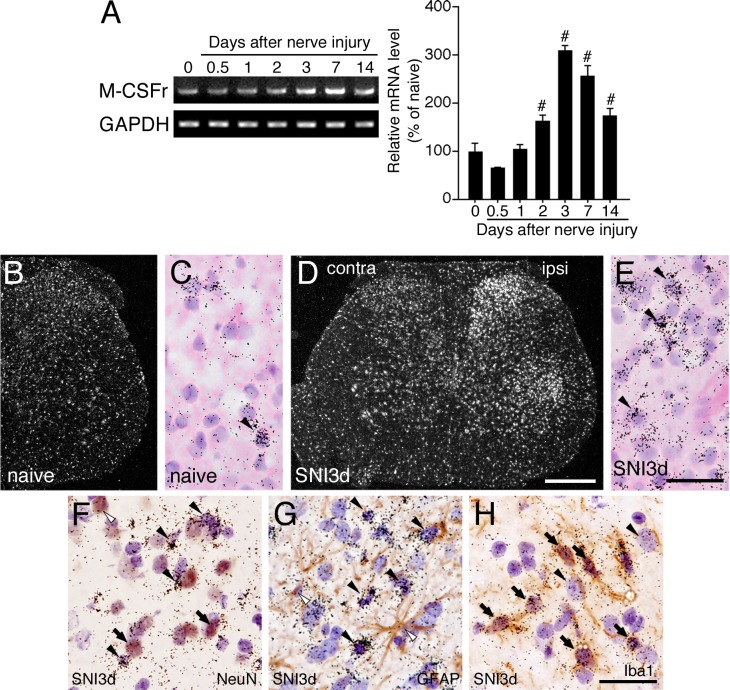
M-CSF receptor mRNA robustly induced in spinal microglia after peripheral nerve injury. PCR products from the spinal cord (L4-5) taken from 0 (naive), 0.5, 1, 2, 3, 7, and 14 days after SNI. Representative gel panels are shown in A. M-CSFr mRNA levels was normalized against GAPDH (*n* = 4, mean ± SEM; #, *p* < 0.05 compared with naive). Darkfield ISHH-images show the expression of M-CSFr mRNA (B, D) in spinal cord of naive (B) and 3 days after nerve injury (D). (C, E) Higher magnification brightfield images of dorsal horn of the left-hand photographs. Sections were counterstained by H-E. (F-H) Double labeling analysis of M-CSFr mRNA at 3 days after SNI. Photographs show combined ISHH for M-CSFr with NeuN (F), GFAP (G) and Iba1 (H) of the spinal dorsal horn at 3 days after SNI. Arrowheads indicate single-labeled cells by ISHH (aggregation of grains), and open arrowheads indicate single immunostained cells (brown staining). Sections were counterstained by hematoxylin. Arrows indicate double-labeled cells. Scale bar = 500 μm in B, D; 25 μm in C, E-H. contra, contralateral; ipsi, ipsilateral.

We narrowed the candidate down to one receptor, a M-CSFr, for detection in the spinal cord after nerve injury, since M-CSF and IL-34 mRNAs, M-CSFr ligands, were selectively detected in the DRG and spinal cord but not GM- and G-CSF mRNAs (Fig 3). RT-PCR revealed that M-CSFr mRNA was detected in naive spinal cord and dramatically increased from 2 to at least 14 days after SNI (Fig 3A). We next performed ISHH to investigate the expression pattern of M-CSFr in the spinal cord following peripheral nerve injury (Fig 3B–3E). Consistent with the results of RT-PCR, darkfield image of ISHH revealed that M-CSFr mRNA was induced in the ipsilateral spinal cord after nerve injury, compared to naive (Fig 3B and 3D). Higher magnification ISHH images of the spinal dorsal horn indicated that M-CSFr mRNA was expressed in the cells whose nucleus were densely or lightly stained by hematoxylin in naive rats (Fig 3C). After nerve injury, M-CSFr mRNA was induced in the cells of dorsal horn, which were densely stained by hematoxylin at 3 days after nerve injury (Fig 3E). We next conducted double-labeling analysis of ISHH with IHC to identify the precise expression of M-CSFr mRNA in the ipsilateral dorsal horn after nerve injury (Fig 3F–3H). Double labeling experiments revealed that expression of M-CSFr mRNA in the spinal cord at 3 days after nerve injury occurred in a subpopulation of NeuN and mainly in Ibal, but not GFAP-ir cells (Fig 3F–3H). These results indicate that M-CSFr mRNA is constitutively detected in a small subset of dorsal spinal neurons and is dramatically induced in ipsilateral spinal microglia after nerve injury.

### M-CSFr inhibition significantly suppressed mechanical allodynia and microglial proliferation induced by peripheral nerve injury in the spinal dorsal horn

**Fig 4:**
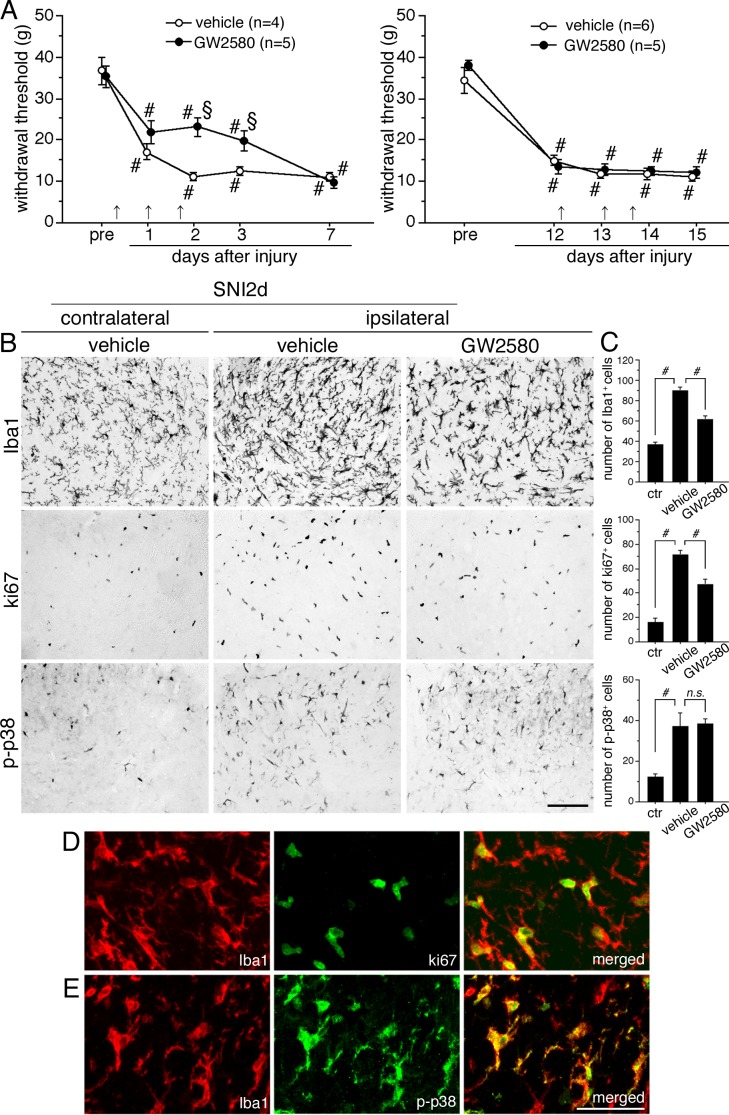
M-CSFr inhibitor suppressed mechanical allodynia in early phase of neuropathic pain and microglial proliferation of spinal dorsal horn after peripheral nerve injury. (left in A) In early phase, the GW2580 (100 nmol, i.t.) was given 3 times at same time, 25 and 40 hours after SNI as arrows shown in bottom. Whereas GW2580 had no effect on mechanical allodynia at 1 day after SNI, mechanical allodynia from 2 to 3 days after SNI was partially but significantly blocked by the repetitive administration of GW2580. (right in A) In late phase, the GW2580 (100 nmol, i.t.) was given 3 times at 12 and 13 and 13 2/3 days after SNI as arrows shown in bottom. Repetitive injection of GW2580 had no effect on the late phase of neuropathic pain after peripheral nerve injury. Open circles indicate vehicle treated animals, solid circles indicate GW2580 treated animals. Data represent as mean ± SEM; *n* = 4–6 per group; #, *p* <0.05 compared with pretreatment; §, *p* < 0.05 compared with vehicle animals at 2 days after SNI. (B) Iba1 (upper lane), ki67 (middle lane) and p-p38 (bottom lane) immunostaining in dorsal horn (lamina I-III) in the contralateral and ipsilateral side of vehicle treated animals and ipsilateral side of GW2580 treated animals at day 2 after SNI. (C) Graphs show quantification of the number of iba1, ki67 and p-p38 positive cells. (D, E) Double immunofluorescence shows that ki67 (green in D) and p-p38 (green in E) are predominantly colocalized with Iba1 (red in D, E) positive cells in spinal dorsal horn of vehicle treated animals at day 2. *n* = 4 per group; #, *p* <0.05; ctr, contra; Scale bar: 100 μm in B; 25 μm in D, E.

Given that M-CSF increased in injured-DRG neurons and its receptor was induced mainly in spinal microglia after peripheral nerve injury, we performed behavioral testing to determine the possible role of M-CSF in the neuropathic pain mechanisms. We examined whether the M-CSFr inhibitor, GW2580, could ameliorate mechanical allodynia in both early and late phase of neuropathic pain (Fig 4A). Since GW2580 was recommended to be used in the solutions on the same day with dilution, we employed the repetitive intrathecal injection method. In early phase, the GW2580 (100 nmol, i.t.) was given 3 times at the same time with SNI surgery, 25 and 40 hours after SNI as arrows indicate at the bottom of the figure (left in Fig 4A). Intrathecal delivery of GW2580 significantly suppressed mechanical allodynia from 2 to 3 days after SNI (left in Fig 4A). In late phase, the GW2580 (100 nmol, i.t.) was given 3 times at 12 (immediate after behavior testing) and 13 and 13 2/3 days (8 hours before behavioral testing) after SNI as arrows indicate at the bottom of the figure (right in Fig 4A). Injection of GW2580 had no effect on the late phase of neuropathic pain after peripheral nerve injury. These results led us to investigate whether intrathecal injection of GW2580 had effects on proliferation and activation of microglia in the spinal dorsal horn in early phase of neuropathic pain, 2 days after SNI (Fig 4B). Since it is known that peripheral nerve injury induced increase, proliferation and activation of microglia [26, 30], we confirmed whether Iba1 (microglia maker), ki67 (proliferating cell maker) and p-p38 positive cells are induced in dorsal horn after SNI (Fig 4B and 4C). Consistent with previous reports, Iba1, ki67 and p-p38 positive cells drastically increased on the ipsilateral side of spinal cord at 2 days after SNI compared to the contralateral side (Fig 4B and 4C). Interestingly, application of GW2580 significantly suppressed the number of Iba1 and ki67 positive cells in dorsal horn of the ipsilateral spinal cord at 2 days after SNI. In contrast, GW2580 had no effect on p-p38 in the spinal dorsal horn after SNI (lower lane in Fig 4B and 4C). Furthermore, we examined whether the expression of ki67 and p-p38 occurs in microglia (Iba1 positive cells) after SNI (Fig 4D and 4E). Iba1 positive cells (red in 4D, E) were heavily colocalized with ki67 (green in Fig 4D) and p-p38 (green in Fig 4E) in the spinal dorsal horn at 2 days after SNI (Fig 4D and 4E). These results suggest that M-CSFr is involved in microglial proliferation in the spinal cord and the early phase of neuropathic pain after peripheral nerve injury.

### Intrathecal administration of M-CSF induced proliferation and activation of microglia in the spinal cord

**Fig 5:**
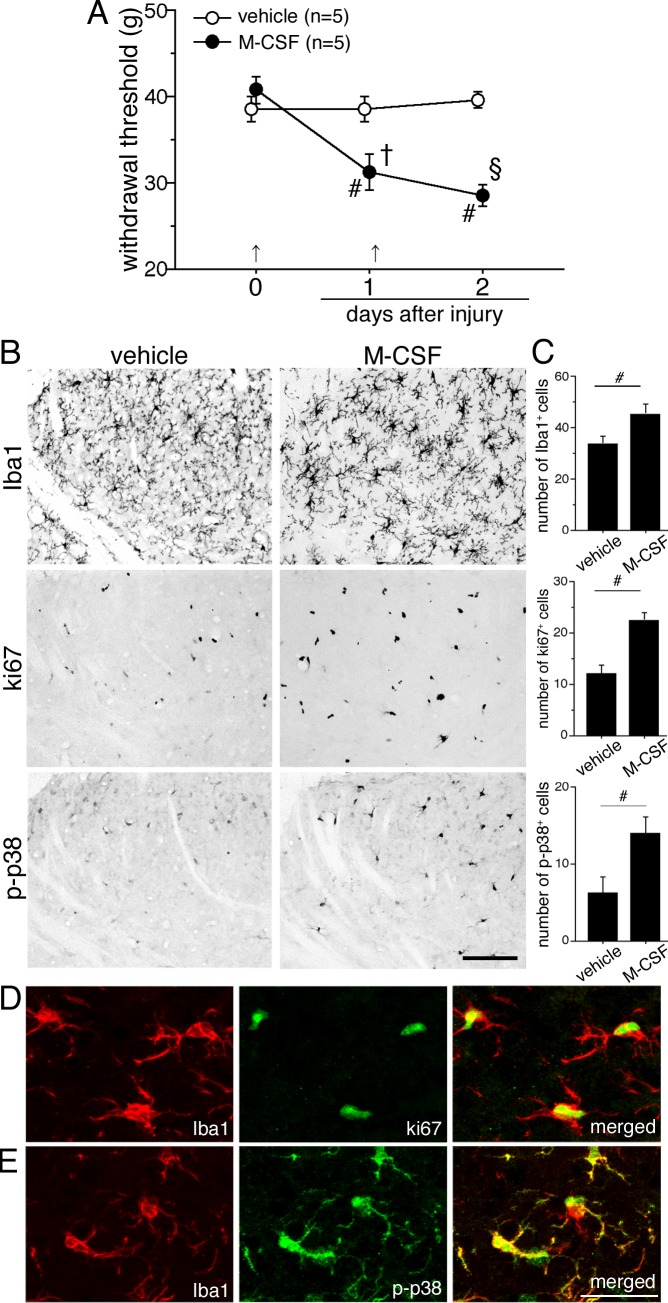
Repetitive intrathecal administration of M-CSF induced mechanical hypersensitivity and microglial proliferation of spinal dorsal horn. (A) The recombinant M-CSF (10 μg, i.t.) was given twice at a 24 h interval, and the behavioral testing was performed 24 h after the each injection. The arrows shown in bottom are time point of injections. Open circles indicate vehicle treated animals (*n* = 5), solid circles indicate recombinant M-CSF treated animals (*n* = 5). #, *p* < 0.05 compared with pretreatment; †, *p* < 0.05 compared with vehicle animals at 1 day after injection, §, *p* < 0.05 compared with vehicle animals at 2 days after injection. Data represent as mean ± SEM; *n* = 5 per group. (B) Iba1 (upper lane), ki67 (middle lane) and p-p38 (bottom lane) immunostaining in dorsal horn (lamina I-III) vehicle and recombinant M-CSF treated animals. (C) Graphs show quantification of the number of iba1, ki67 and p-p38 positive cells. (D, E) Double immunofluorescence shows that ki67 (green in D) and p-p38 (green in E) are predominantly colocalized with Iba1 (red in D, E) positive cells in spinal dorsal horn of M-CSF treated animals at day 2. Data represent as mean ± SEM; *n* = 4 per group. #, *p* < 0.05 compared with vehicle;Scale bar: 100 μm in B; 25 μm in D, E.

We asked whether M-CSF itself leads to mechanical allodynia, we intrathecally injected recombinant M-CSF (Fig 5A). The recombinant M-CSF (10 ug, i.t.) was given twice at a 24 h interval, and the behavioral testing was performed 25 h after each injection. Intrathecal delivery of M-CSF significantly reduced mechanical threshold of the hindpaw at 1 day after the first injection (Fig 5A). We next wanted to know whether intrathecal injection of M-CSF has effects on microglial proliferation and activation in spinal dorsal horn (L4-5). Thus, we performed IHC to examine the number of Iba1, ki67 and p-p38 positive cells in the spinal dorsal horn (Fig 5B and 5C). As expected, injection of M-CSF increased the number of Iba1 and ki67 positive cells in the spinal dorsal horn (upper and middle lane in Fig 5B and 5C). In contrast to the result in Fig 4, the induction of p-p38 occurred in the spinal dorsal horn following M-CSF injection (lower lane in Fig 5B and 5C). Finally, to determine whether M-CSF induced-proliferation occurs in microglia, a double immunofluorescence study was performed (Fig 5D and 5E). It showed that ki67 (green in Fig 5D) and p-p38 (green in Fig 5E) after M-CSF injection are induced in Iba1 positive cells (red in Fig 5D and 5E). These results suggest that M-CSF is one of the key molecules which induce microglial proliferation in the spinal cord *in vivo* and neuropathic pain-like behaviors.

## Discussion

CSFs are well known as hematopoietic cytokines that regulate survival, differentiation and proliferation of hematopoietic linages. G- and GM-CSF have often been applied to patients with acute radiation syndrome after irradiation due to carcinoma [31]. Recently, nonhematopoietic effects of CSFs have been reported, including an effect on the peripheral tissues. G- and GM-CSF were produced by hindpaw bearing cancer, leading to the development of cancer pain (18). In the immediate past, it has been reported that M-CSF protein was upregulated in injured-primary afferent after peripheral nerve injury in mice [19]. However, in terms of rat DRGs, there are no report which investigate the expression of a series of CSFs and IL-34. Here, we show that M-CSF, but not G- and GM-CSF mRNA, is weakly expressed in the naive DRG and robustly increases in DRG neurons which were labeled with ATF3-ir cells. Size distribution analysis revealed that induction of M-CSF mRNA widely occurred through the small neurons to the largest ones. Furthermore, M-CSF mRNA did not change in DRG of CFA model rats in which limited activation of spinal microglia is induced. These findings indicated that increased expression of M-CSF in primary afferents was dependent on “direct”-nerve injury. This increase of M-CSF mRNA occurred within 1 day after nerve injury indicating a rapid onset of induction. These results strongly support the data which was reported by Guan et al [19].

A previous study showed that M-CSF increased in the brain of patients with Alzheimer’s disease (AD), brain tumor and in several brain injury models [32–34]. In the brain of the AD model, M-CSF increased in neurons near the accumulation of beta-amyloid [35]. It has been reported that M-CSF was upregulated in neurons of the striatum which were injured by injection of ethanol [36]. Furthermore, M-CSF increased in neurons of the hippocampus after kainic acid induced-brain injury [37]. This accumulating evidence strongly suggests that M-CSF is increased in injured neurons and plays a role in the pathological events after nerve injury. It has been demonstrated that M-CSFr is specifically expressed by microglia in the brain [38, 39]. It has been reported that M-CSFr was expressed and increased in microglia around the facial nucleus after facial nerve injury [17, 39]. Furthermore, they demonstrated that bath application of recombinant M-CSF apparently caused proliferation of primary cultured microglia *via* induction of proliferation cell nuclear antigen *in vitro* [17].

Previous report suggested that the induction of migration or proliferation of macrophage in injured fibers of primary afferent occurred from 3 days after nerve injury [40]. It is well known that peripheral nerve injury triggers the proliferation/activation of microglia in the spinal cord at day 3 [41, 42]. Our findings in the context of previous studies suggest that increased M-CSF is released from the peripheral and central terminal of primary afferent leading to proliferation of macrophage in the periphery and microglia in the spinal cord after nerve injury. In terms of microglial proliferation, it is known that the increase of microglia in the spinal cord after peripheral nerve injury is due to proliferation, because the number of BrdU expressing cells increased in spinal microglia after peripheral nerve injury [26, 43]. However, it has been reported that disruption of the blood brain barrier following peripheral nerve injury induces infiltration of circulating monocytes into the spinal cord parenchyma and these cells become Iba1-positive cells, like resident microglia [44]. Hence, two mechanisms, proliferation of resident microglia and infiltration of circulating cells into spinal cord after peripheral nerve injury, have been demonstrated. Since our results indicated that intrathecal injection of M-CSF caused the proliferation of spinal microglia, M-CSF derived from injured-primary afferents may induce the proliferation of “resident microglia” in the affected segment of spinal cord after peripheral nerve injury *in vivo*.

Activated microglia are also known to release many proinflammatory molecules and neurotrophic factors, such as IL-1ß, IL-6, TNF-alpha and brain-derived neurotrophic factor (BDNF) [45, 46]. These cytokines are generally produced *via* p-p38 MAPK in activated microglia and are involved in pain hypersensitivity [47]. In the present study, intrathecal injection of recombinant M-CSF induced p-p38 in microglia (lower lane in Fig 5B and 5E) and mechanical hypersensitivity (Fig 5A). We propose that M-CSFr signaling through p-p38 MAPK in activated microglia is a trigger for the production of proinflammatory cytokines and contributes to mechanical hypersensitivity. However, in early phase of neuropathic pain, M-CSFr inhibition did not have an effect on p-p38 in the dose that reversed the mechanical allodynia (Fig 4). A plausible explanation of this discrepancy is that induction of p-p38 in microglia is not only due to M-CSFr but also due to many other receptors, such as P2Y12 and CX3CR1 [48, 49]. We also considered that p-p38 might not be a primary downstream product of M-CSFr, or that M-CSFr has the signaling cascades that are independent of p-p38 pathway after peripheral nerve injury. Whereas Guan et al. [19] recently demonstrated that M-CSF depletion in DRG neuron could continuously reversed mechanical allodynia, in our experiment M-CSFr inhibitor had a transient ameliorative effect on mechanical allodynia. This discrepancy may be due to method (cKO vs pharmacological inhibition) or species (mice vs rats). As shown in right side in Fig 4A, the delayed administration of GW2580 did not reverse mechanical allodynia from 13 to 15 days after peripheral nerve injury. Since it has been reported that microglial proliferation in spinal cord occurred within 3 days after peripheral nerve injury [26], we convinced the reason why M-CSFr inhibitor was not effective during late phase of neuropathic pain is that the effects of activated microglia disappeared at this point. IL-34 has been recently identified as an alternative ligand for M-CSFr [50], but there is limited evidence about the expression. The expression of IL-34 was reported in keratinocytes [51] and the deficient of IL-34 decreased the number of Langerhans cells in the epidermis [52]. The present study shows that IL-34 mRNA is constitutively expressed in non-neuronal cells of the DRG and is not affected by peripheral nerve injury. We anticipated that it localized in the satellite cells around DRG neurons due to the location and staining intensity of hematoxylin. Given that M-CSFr is predominantly expressed in mononuclear phagocyte lineage cells, such as monocytes, macrophages, Langerhans cells and microglia [53], IL-34 in the DRG may play a role in maintenance of the number of macrophages around the DRG. Our findings revealed that IL-34 was expressed in a subpopulation of dorsal horn neurons and had no change in response to nerve injury. Interestingly, in IL-34 deficient reporter mice it was clearly shown that neurons are a predominant source of IL-34 and that the number of microglia was reduced compared with wildtype mice [54]. Our current observations in the context of previous findings may indicate that IL-34 produced by dorsal horn neurons consistently maintains the number of microglia and have no effect on proliferation of microglia in the spinal cord after nerve injury.

In contrast to expression of M-CSF and IL-34 mRNA, GM- and G-CSF could not be detected in the DRG and spinal cord of both naive mice and in the SNI model as shown in Figs 1A and 2A. Although there is no report that GM- and G-CSF were expressed in DRG and spinal cord, we tested whether the primers in this study were available. Fig 1B indicated that each primer should be available, because precise bands were generated in the thymus as a positive control. And for ISHH, we confirmed amplified fragments are correct by DNA sequencing (data not shown). Therefore, we concluded that GM- and G-CSF mRNAs were not expressed or detectable in the DRG and spinal cord.

## Conclusions

M-CSF, derived from injured-primary afferents, induced a microglial proliferation in the spinal cord after peripheral nerve injury *in vivo*, and proliferation of microglia induced by M-CSF leads to the generation of neuropathic pain in rats. Our data is strongly consistent with the data in mice that was recently reported by Guan et al [19]. Accumulating evidence suggests that the appropriate management of the number of microglia in the spinal cord through M-CSF may be one of useful avenue towards the development of novel therapies for neuropathic pain following peripheral nerve injury.
